# Increased Risk of Infertility in Women Infected with Human Papillomavirus

**DOI:** 10.18502/jri.v24i3.13275

**Published:** 2023

**Authors:** Parastou Heidari Pebdeni, Fereshteh Saffari, Hamid Reza Mollaei, Toraj Reza Mirshekari, Robabeh Hosseini Sadat, Victoria Habibzadeh, Lida Saeed, Moslem Taheri Soodejani, Roya Ahmadrajabi

**Affiliations:** 1- Student Research Committee, Kerman University of Medical Sciences, Kerman, Iran; 2- Department of Medical Microbiology (Bacteriology & Virology), Afzalipour Faculty of Medicine, Kerman University of Medical Sciences, Kerman, Iran; 3- Afzalipour Clinical Center for Infertility, Kerman University of Medical Sciences, Kerman, Iran; 4- Department of Obstetrics and Gynecology, School of Medicine, Kerman University of Medical Sciences, Kerman, Iran; 5- Center for Healthcare Data Modeling, Department of Biostatistics and Epidemiology, School of Public Health, Shahid Sadoughi University of Medical Sciences, Yazd, Iran; 6- Medical Mycology and Bacteriology Research Center, Kerman University of Medical Sciences, Kerman, Iran

**Keywords:** Female, Infertility, Iran, Sexually transmitted infections

## Abstract

**Background:**

Among several causes of infertility, urogenital infections seem to be influencing factors. The effect of bacterial or viral sexually transmitted infections (STIs) on human fertility is not well understood. The aim of this study was to determine the frequency of STIs in cervical samples of infertile and fertile women and study the relationship between these agents and infertility.

**Methods:**

In this case-control study, cytobrush was used for collecting of cervical sample from each infertile and fertile woman (n=95) who attended Research and Clinical Centers for Infertility in Kerman, Iran. PCR and real-time PCR methods were used to detect the presence of bacterial (genital *Ureaplasma* species, genital *Mycoplasma* species, *Chlamydia trachomatis (C. trachomatis)*, and *Gardnerella vaginalis*) and viral (herpes simplex virus, human papillomavirus (HPV), and Epstein-Barr virus) agents, respectively. Fisher’s exact test and the logistic regression with the significance level of ≤5% were used for statistical analyses.

**Results:**

In general, 78.94% and 14.73% of specimens were positive for one or more studied *microorganisms*, respectively. Among studied agents, only the infection with HPV was significantly different between infertile and fertile groups (p=0.005) which may enhance the likelihood of female infertility (OR=5.30, 95% CI:1.47–19.11, p< 0.05). After adjusting for age, irregular menstrual cycle, abnormal vaginal discharge, and ectopic pregnancy, the odds ratio of infertility in HPV-infected women increased (OR=7.02, 95% CI:1.52–32.3, p<0.05).

**Conclusion:**

Since HPV infection is asymptomatic, periodic screening of women in reproductive age especially infertile couples is recommended for early diagnosis and prevention of infection progression and cross contamination.

## Introduction

Infertility is a prevalent problem affecting almost 15% of couples worldwide (1). In Iran, the overall prevalence of infertility has grown to 7.88% (95% CI: 5.61–10.51), and approximately 21–22% of Iranian women suffer from infertility (2). Urogenital infections seem to be one of the major causes of infertility (1). More than 30 bacterial, viral or parasitic agents are responsible for these infections (3). Chlamydia, herpes simplex virus (HSV), and human papillomavirus (HPV) are the most prevalent sexually transmitted infections (STIs) (4). Since urogenital infections are often either asymptomatic or mild symptomatic, they may not be diagnosed by patients and clinicians (5). Long-term infection can lead to severe reproductive health complications in women, such as stillbirth, preterm delivery, increased risk of HIV acquisition, infertility, cancer, and so on (4). Untreated *Chlamydia trachomatis (C. trachomatis)* infection can lead to pelvic inflammation which causes scars inside the reproductive organs and consequently may affect fertility (6). Genital *Mycoplasma* and *Ureaplasma* infections can ascend into the upper genital tract and colonize the endometrium, fallopian tubes, and ovaries which lead to major long-term consequences including tubal damage, impairment in oocyte transport, and ultimately infertility (7). *Gardnerella vaginalis* is an anaerobic bacterium that resides in the normal vaginal flora. Although *Gardnerella* is not considered to be contagious, its transmissibility is yet to be completely understood. The spread of this bacterium among individuals through sexual intercourse may alter the natural balance of *microorganisms* inside the vagina and can even lead to the development of bacterial vaginosis (BV), which might increase the risk of infertility and STIs, including infections with Chlamydia, Gonorrhea, and HPV (8, 9). Alterations of reproductive functions may be linked to viral STIs including HSV, HPV, human cytomegalovirus, human immunodeficiency virus, as well as adeno-associated viruses. The effect of viral STIs on human fertility is not well understood (10). HPV infections have been displayed to be substantially linked to many adverse effects on reproduction function that may affect fertility. If a woman is HPV-positive, the rate of premature ruptures of membranes and spontaneous abortion would be higher due to the feasible transition of the virus to oocytes during fertilization which impact or induce the immune system response (11). Asymptomatic reproductive tract infections might be etiologic factors in unexplained infertility (12). To the best of our knowledge, no adequate research was performed on the frequencies of simultaneous bacterial and viral infections in infertile and fertile women in Kerman (southeast of Iran). Consequently, the purpose of this study was to determine the frequency of sexually transmitted bacteria and viruses as well as their simultaneous presence in cervical samples of infertile and fertile women and study the relationship between the presence of these agents and infertility using the molecular assays.

## Methods

### Collection of sample:

From March to August 2020, using random sampling, a total of 95 infertile women aged 20 to 49 years who attended the Research and Clinical Centers for Fertility in Kerman were included in this case-control study. Infertility was defined as “a failure to conceive after at least 12 months of unprotected sexual intercourse” (13). Infertility can be primary or secondary. Primary infertility is defined as inability to conceive within two years of regular unprotected intercourse (*i.e*. being sexually active, nonlactating, and not being on contraception) among 15 to 49-year-old women. Secondary infertility refers to the inability to conceive following a previous pregnancy (14).

The selection criteria for the participants were as follows: a) non-male factor infertility, b) lack of hormonal and reproductive system abnormalities (hyperprolactinemia, anovulation, tubal diseases, premature ovarian failure, abnormalities of the cervix, endometriosis, luteal phase defects, and other endocrine or medical disorders), and c) lack of infection symptoms in the lower genital tract (12, 15). None of the patients was under treatment with local or systemic antibacterial and antiviral medication in the last two weeks and they had sexual abstinence for 48 *hr* (15). The control group consisted of 95 women in the similar age range to the infertile group, with a history of giving birth to at least one child as well as the absence of inflammation, benign ovarian tumors, and uterine myomas (16). Informed consent was obtained from all participants. Moreover, the present study was approved by Ethics Committee of Research Council of Kerman University of Medical Sciences, Kerman, Iran (IR.KMU.REC.1399.282).

All subjects completed questionnaires, including questions on age, dyspareunia, dysmenorrhea, amount of bleeding, irregular menstrual cycle, post coital bleeding (PCB), abnormal vaginal discharge, infertility type, histories of gravidity, abortion, ectopic pregnancy, and surgery.

Sampling was performed by a gynecologist using sterile cervical cytobrush to collect tissues from the cervix of each woman under sterile conditions. The cytobrush was then placed in 2 *ml* of liquid based transport media and frozen at −20°*C* for polymerase chain reaction (PCR) assay. All cervical samples were subjected to bacterial and viral DNA extraction using nucleic acid extraction kit (AmpliSens^®^ RIBO-prep; Interlabservis, Russia) based on manufacturer’s protocol. The quality of the extracted DNA was confirmed by measuring the absorption of DNA at 260 *nm* and 280 *nm* using NanoDrop ND 1000 spectrophotometer (NanoDrop Technologies, USA).

### Molecular detection:

PCR assay was used to determine the presence of *Ureaplasma* spp., *Ureaplasma urealyticum (U. urealyticum)*, *Ureaplasma parvum (U. parvum)*, *Mycoplasma hominis (M. hominis)*, and *Gardnerella vaginals (G. vaginalis)* by amplification of ure A–B gene, specific multiple banded antigen (MBA) for *U. urealyticum* or *U. parvum*, specific 16S rRNA for M. hominis or *G. vaginalis* as described previously (13, 17).

### Detection of Mycoplasma genitalium by semi-nested PCR:

The first conventional PCR was carried out for the amplification of 286-*bp* adhesion DNA fragment using F1: AGTTGATGAAACCTTAA CCCCTTGG and R1: CCGTTGAGGGGTTTTC CATTTTTGC primers. Semi nested-PCR was then employed to amplify 194-*bp* adhesion DNA fragment using two specific primers (F1: AGTTGATGAAACCTTAACCCCTTGG and R2: GACC ATCAAGGTATTTCTCAACAGC) as described previously (13). Previously sequenced clinical isolates of each bacterium were used as positive controls (13).

### Detection of Chlamydia trachomatis:

For *C. trachomatis*, PCR was performed based on detection kit’s instructions (Iranian Gene Fanavar Institute, Iran).

### Detection of viruses:

Real-time PCR experiments were carried out in an ABI StepOnePlus Real-time PCR system with EvaGreen PCR Master Mix (Applied Biosystems, USA) to detect HSV-1, HSV-2, HPV, and Epstein-Barr virus (EBV) in the samples (18–20). The positive control for each virus was obtained from Department of Microbiology and Virology of Kerman University of Medical Sciences, Kerman, Iran.

### Statistical analysis:

Data analysis was done using SPSS Statistics version 16 (IBM, USA). Descriptive statistics including frequency was used for categorical variables. Fisher’s exact test was employed to assess the relationship between the prevalence of some studied *microorganisms* and infertility. The logistic regression was conducted to predict the effect of risk factors on women’s infertility. The significance level of ≤5% was regarded in all statistical analyses.

## Results

The analysis was performed on 190 cervical cytobrush samples. Infertile women aged 20–49 years (30.96±6.94) and fertile women 20–50 years (34.27±7.1).

Analysis of reproductive and gynecological characteristics in infertile group showed that 62.1% (n=59) and 37.9% (n=36) of women suffered from primary and secondary infertility, respectively. No significance difference was found in most of the studied parameters, as shown in table 1, between two groups of infected and uninfected infertile women. But in some variables such as irregular menstrual cycle and surgery, sample size or other factors may affect the results; therefore, no definite interpretation can be concluded on these characters.

**Table 1.** Reproductive and gynecological characteristics in infected and uninfected infertile women

**Variables**	**Frequency (%)**		**p-value**

	**Infected (n=60)**	**Uninfected (n=35)**	
**Gravidity**			
Positive	21 (35)	12 (34.3)	1.000
Negative	39 (65)	23 (65.7)	
**Abortion**			
Positive	10 (16.7)	6 (17.1)	1.000
Negative	50 (83.3)	29 (82.9)	
**History of ectopic pregnancy**			
Positive	1 (1.7)	1 (2.9)	1.000
Negative	59 (98.3)	34 (97.1)	
**Dyspareunia**			
Positive	10 (16.7)	7 (20)	0.783
Negative	50 (83.3)	28 (80)	
**Dysmenorrhea**			
Positive	12 (20)	11 (31.4)	0.225
Negative	48 (80)	24 (68.6)	
**Amount of bleeding**			
Normal	1 (1.7)	2 (5.7)	0.552
Abnormal	59 (98.3)	33 (94.3)	
**Irregular menstrual bleeding**			
Regular	11 (18.3)	11 (31.4)	0.207
Irregular	49 (81.7)	24 (68.6)	
**Post coital bleeding**			
Positive	1 (1.7)	0 (0)	1.000
Negative	59 (98.3)	35 (100)	
**Abnormal vaginal discharge**			
Positive	26 (43.33)	16 (45.71)	0.834
Negative	34 (56.66)	19 (54.28)	
**Infertility type**			
Primary	38 (63.3)	21 (60)	0.828
Secondary	22 (36.7)	14 (40)	
**Surgical history**			
Positive	12 (20)	12 (34.3)	0.146
Negative	48 (80)	23 (65.7)	

In general, 78.94% of specimens (150/190) were positive for one or more studied bacterial agents. Overall, 45.78% of total women carried *Ureaplasma* spp., of whom 23.15% carried U. parvum (serovars 1, 3, 14), 6.84% *U. urealyticum*, and the remained ones carried the other serovars which were not detectable by used primers (Table 2).

**Table 2.** Association between the presence of *Mycoplasma* genitalium, *Mycoplasma* hominis, *Ureaplasma* spp., *Ureaplasma* urealyticum, *Ureaplasma* parvum, *Chlamydia trachomatis*, *Gardnerella vaginalis*, human papillomavirus, Epstein-Barr virus, human herpesvirus-1, and human herpesvirus-2 in cervical cytobrush specimens of fertile and infertile women

**Bacteria**	**Positive number (%)**			**p-value**

	**Infertile**	**Fertile**	**Total**	
***M. genitalium***	6 (6.31)	2 (2.1)	8 (4.21)	0.279
***M. hominis***	3 (3.15)	5 (5.26)	8 (4.21)	0.721
***Ureaplasma* spp.**	38 (40)	49 (51.57)	87 (45.78)	0.109
***U. urealyticum***	8 (8.42)	5 (5.26)	13 (6.84)	0.389
***U. parvum***	24 (25.26)	20 (21.05)	44 (23.15)	0.492
***G. vaginalis***	20 (21.05)	24 (25.26)	44 (23.15)	0.492
***C. trachomatis***	3 (3.15)	0 (0)	3 (1.57)	0.246
**Human papillomavirus**	14 (14.73)	3 (3.15)	17 (8.94)	0.005
**Epstein-Barr virus**	3 (1.57)	0 (0)	3 (1.57)	0.246
**Human herpesvirus-1**	2 (2.1)	0 (0)	2 (1.05)	0.497
**Human herpesvirus-2**	5 (5.26)	1 (1.05)	6 (3.15)	0.211

There was no statistically significant difference in the presence of each bacterium in infertile women versus fertile ones (Table 2). A high rate of co-infection with *Ureaplasma* spp. and G. vaginalis was observed in the infertile and fertile groups. The highest frequency of simultaneous infection with *U. parvum* and *U. urealyticum* was seen in infertile women (6.31%, n=6). Simultaneous infection of *U. parvum* and *G. vaginalis* was only observed in 5.26% (n=5) of the fertile group (Table 3).

**Table 3.** Frequency of co-existence of tested bacteria in cervical cytobrush specimens of fertile and infertile women

**Bacteria**	**Positive number (%)**	

	**Fertile**	**Infertile**
***Ureaplasma* spp. + *M. hominis***	4 (4.21)	2 (2.1)
***U. urealyticum* + *M. hominis***	1 (1.05)	0 (0)
***U. parvum* + *M. hominis***	3 (3.15)	2 (2.1)
***U. parvum* + *U. urealyticum***	3 (3.15)	6 (6.31)
***M. hominis* + *M. genitalium***	1 (1.05)	0 (0)
***Ureaplasma* spp. + *M. genitalium***	2 (2.1)	0 (0)
***U. parvum* + *M. genitalium***	1 (1.05)	0 (0)
***U. urealyticum* + *M. genitalium***	1 (1.05)	0 (0)
***M. genitalium* + *G. vaginalis***	0 (0)	1 (1.05)
***M. hominis* + *G. vaginalis***	2 (2.1)	2 (2.1)
***C. trachomatis* + *M. hominis***	0 (0)	1 (1.05)
***Ureaplasma* spp. + *C. trachomatis***	0 (0)	2 (2.1)
***Ureaplasma* spp. + *G. vaginalis***	12 (12.63)	13 (13.68)
***U. urealyticum* + *G. vaginalis***	1 (1.05)	3 (3.15)
***U. parvum* + *G. vaginalis***	5 (5.26)	0 (0)
***Ureaplasma* spp. + *M. genitalium* +*M. hominis***	1 (1.05)	0 (0)
***M. hominis* + *M. genitalium* + *U. parvum***	1 (1.05)	0 (0)
***M. hominis* + *M. genitalium* + *U. urealyticum***	1 (1.05)	0 (0)
***M. hominis* + *Ureaplasma* spp. + *G. vaginalis***	2 (2.1)	0 (0)
***C. trachomatis* + *U. parvum* + *G. vaginalis***	0 (0)	1 (1.05)
***U. parvum* + *C. trachomatis* + *G. vaginalis***	0 (0)	1 (1.05)

*M. hominis: Mycoplasma hominis, M. genitalium: Mycoplasma genitalium, U. parvum: Ureaplasma parvum, U. urealyticum: Ureaplasma urealyticum, C. trachomatis: Chlamydia trachomatis, G. vaginalis: Gardnerella vaginalis*

In general, 14.73% of specimens (28/190) were positive for one or more studied viral agents. As reported in table 2, among infertile women, HPV was the most frequent problem. There was only a significant difference in the presence of HPV between infertile and fertile females (p=0.005). Concomitant viral infection was not observed in any of the fertile and infertile groups.

In the fertile group, out of 3 women infected with HPV, one had a simultaneous infection with *M. hominis*, *M. genitalium*, and *U. urealyticum* and one had a simultaneous infection with *M. hominis*, *U. parvum*, and *G. vaginalis*. Concurrent infection with HSV-2 and *Ureaplasma* spp. was observed in one HSV-2 positive woman. In the infertile group, co-infection with HPV and *Ureaplasma* spp. (5.26%, n=5) and HPV with *G. vaginalis* (4.2%, n=4) was predominant. Table 4 summarizes the bacterial co-infections with HPV and other viruses detected in infertile women.

**Table 4.** Frequency of co-existence of tested bacteria and viruses in infertile specimens

***Microorganisms***	**Number (%)**
**HPV + *C. trachomatis***	1 (1.05)
**HPV + *M. genitalium***	3 (3.15)
**HPV + *G. vaginalis***	4 (4.2)
**HPV + *Ureaplasma* spp. **	5 (5.26)
**HPV + *U. parvum***	2 (2.1)
**HPV + *U. urealyticum***	1 (1.05)
**HPV + *M. genitalium* + *G. vaginalis***	1 (1.05)
**HPV + *U. parvum* + *G. vaginalis***	1 (1.05)
**HPV + *U. urealyticum* + *G. vaginalis***	1 (1.05)
**HSV-1 + *M. genitalium***	1 (1.05)
**HSV-1 + *Ureaplasma* spp. **	1 (1.05)
**HSV-2 + *Ureaplasma* spp. **	2 (2.1)
**HSV-2 + *G. vaginalis***	1 (1.05)
**HSV-2 + *U. parvum***	1 (1.05)
**HSV-2 + *U. urealyticum***	1 (1.05)
**HSV-2 + *G. vaginalis* + *U. parvum* + *U. urealyticum***	1 (1.05)

*M. genitalium: Mycoplasma genitalium, U. parvum: Ureaplasma parvum, U. urealyticum: Ureaplasma urealyticum, C. trachomatis: Chlamydia trachomatis, G. vaginalis: Gardnerella vaginalis*, HPV: human papillomavirus, HSV-1: human herpesvirus-1, HSV-2: human herpes-virus-2

Univariate analysis indicated that HPV infection could increase the odds of infertility in women (OR=5.30, 95%CI: 1.47–19.11) and the odds for infertility in infected women with HPV was 5.30 times higher than in uninfected ones. After adjusting for age, irregular menstrual cycle, abnormal vaginal discharge, and ectopic pregnancy, the odds ratio of infertility in women infected with HPV increased (OR=7.02, 95% CI:1.52–32.3, p< 0.05) (Table 5).

**Table 5.** Adjusted and crude odds ratio for relationship between HPV and infertility

**Variables**		**Number of fertile women (%)**	**Number of infertile women (%)**	**Crude OR (95% CI)**	**Adjusted OR (95% CI)**
**Age (year)**	38<	54 (56.8)	23 (24.2)	1.7 (0.082–3.7)	2.3 (0.96–5.3)
	<38	41 (43.2)	72 (75.8)	1	1
**Ectopic pregnancy**	Positive	3 (3.2)	2 (2.1)	0.66 (0.11–4.04)	0.39 (0.06–2.65)
	Negative	92 (96.8)	93 (97.9)	1	1
**Irregular menstrual cycle**	Regular	58 (61.1)	22 (23.2)	0.19 (0.10–0.36) ^**^	0.16 (0.08–0.33) ^**^
	Irregular	37 (38.9)	73 (76.8)	1	1
**Abnormal vaginal** **discharge**	Positive	22 (23.2)	42 (44.2)	2.63 (1.41–4.92) ^**^	2.4 (1.16–4.94) ^*^
	Negative	73 (76.8)	53 (55.8)	1	1
**HPV**	Positive	3 (3.2)	14 (14.7)	5.30 (1.47–19.11) ^*^	7.02 (1.52–32.3) ^*^
	Negative	92 (96.8)	81 (85.3)	1	1

HPV: Human papillomavirus

OR (95% CI): Odd ratio (95% confidence interval).

^*^ p<0.05,

^**^ p<0.01

## Discussion

Currently, the rate of infertility is growing, which, in addition to affecting the quality of life and psychological characteristics of patients, imposes an economic burden on families. Sexually transmitted infections are responsible for 20–60% of female infertility cases (21).

One of the most common worldwide sexually transmitted viruses is the HPV, which is suggested as a fertility-changing factor. HPV infection is associated with chronic inflammation, infertility, and early abortion (22). HPV was detected in 14.6% of infertile and 3.2% of fertile women. In this respect, conflicting results were obtained by different studies conducted in various parts of the world. For instance, Rocha et al. in Brazil found that 60% and 17.1% of the infertile and control group were positive for HPV, respectively (23). In the study of Lundqvist et al., it was shown that 7% of the infertile women were HPV-positive compared with 9.1% of the controls (24). Perino et al. performed a research on infertile couples in Palermo, Italy, and reported that the female partners had a positive HPV DNA test among 17.5% couples (25). There was a substantial difference in the frequency of HPV in the infertile and fertile groups in our research, comparable to Zhang et al.’s study (26). In contrast, in other studies from different countries, no difference was observed between the frequency of HPV in infertile and fertile groups (24, 27). Similar to our study, Yuan et al. showed a significant association between HPV and female infertility (21). Hsu et al. demonstrated that the adjusted hazard ratio of infertility in HPV patients relative to the control patients was 1.39 (22). Although some factors such as the type of study, sample size, statistical analysis, and age range of participants are different between our study and others, HPV can be considered a potential risk factor of female infertility. Since among women with genital HPV, the infection is usually transmitted via contact with infected sexual partner, examination of men, even if they are asymptomatic, is recommended to improve infertility treatment (24).

Other viruses such as EBV, HSV-1, and HSV-2 may be related to viral STIs. Berntsson et al. performed a study on healthy young women and reported frequency of EBV, HSV-1, and HSV-2 in 10.5%, 1.7%, and 1.4% of them, respectively (28), whereas in our study, HSV-2 was found only in 1.05% of fertile females.

The presence of EBV in the women’s genital tract allows the interactions between co-existing EBV and HPV infections (29). However, in our study, co-infection with these viruses was not seen in the infertile group. Epstein-Barr virus is rarely associated with female infertility, and may be linked to altered immunophenotypic factors as well as peripheral immunostimulatory effects in women with a history of infertility (30). Specifically, 1.57% of our studied infertile women were infected with EBV.

The study of Li et al. indicated that the rate of HSV-2 in infertile and normal women was 80.0% and 25.6%, respectively, and there was a significant difference between them (31). In our study, the prevalence was very low (5.26% *vs*. 1.05% for infertile and fertile females, respectively) without a significant difference between the two groups.

Some bacterial agents can be sexually transmitted. *C. trachomatis* is one of the most common sexually transmitted bacteria. In more than 70% of women, genital *chlamydia* infection is asymptomatic, thus making them a reservoir for further transmission (32, 33). The frequency of C. trachomatis genital infection among infertile women who had primary infertility was 3%, much less than the 15.7% reported by Rawre et al. in India (34), 36.2% reported by Ramadhan et al. in Tanzania (35), and 16.6% reported by Haghdoost et al. in Tabriz, Iran (36), while being close to the values reported from Wroclaw, Poland (37), Kashan (38), and Ahvaz from Iran (39). The impact of C. trachomatis infection on female fertility is not completely established yet, since the evidence corroborating the association is relatively weak (40). Studies on the correlation between C. trachomatis and female infertility have manifested conflicting results. Some studies have demonstrated an important association between previous C. trachomatis infection and female infertility (32, 34, 35) while others have shown that infertility may occur independent of this agent (39, 41, 42). Our study was consistent with the latter group of studies as no association was observed between C. trachomatis infection and female infertility. It seems that this result may be due to the low number of women with secondary infertility in the studied population.

In Iran, the prevalence of urogenital *mycoplasmas* in women is high and ranges from 2 to 40.5%. Although the role of urogenital *mycoplasmas* in female infertility is controversial, the effect of *M. genitalium* on infertility, particularly tubal infertility, was shown (43). The prevalence of M. genitalium in our examined infertile women was higher than in a prior study from Kerman, Iran (23.2%) (15), but lower than the value reported by Grześko et al in Poland with a significant difference between fertile and infertile females (16). The frequency of *M. hominis* among the infertile group was 31.6% lower than in a previous report from Kerman (44) and closely similar to the result reported from Tehran, Iran (45). Although the prevalence of *U. urealyticum* in our study was somewhat similar to that of Seifoleslami et al. (45), no significant difference was observed between fertile and infertile groups.

In our study, the frequency of G. vaginalis in infertile and fertile asymptomatic females was somehow similar. In the study of Casari et al. on the genital discharges of asymptomatic women with infertility problems, the most prevalent bacterium was *G. vaginalis* with a frequency of 19.7%. These researchers suggested that *microorganisms* such as *G. vaginalis* should be better studied based on the association found between idiopathic infertility and the presence of cervical cytokines in women with abnormal vaginal flora (46). Different prevalence rates of bacteria or viruses reported by various studies may be related to several factors such as the diversity of the participants, type of samples, number of samples, experimental methods with different sensitivity and specificity, age, socioeconomic conditions, the number of sexual partners, as well as geographical and cultural features of countries (13, 45).

## Conclusion

Since urogenital infections are often asymptomatic, periodic screening by molecular tests is essential. Our results showed that HPV is the most abundant viral agent detected among infertile women, and an association was found between HPV and infertility; therefore, periodic screening by PCR assay is recommended for early diagnosis, prevention of progression of the infection, as well as follow-up studies and large-scale longitudinal studies in other regions to determine the exact role of HPV in infertility.
